# Unleashing potential and optimizing adolescent roller skating performance through a structured exercise program – a randomized controlled trial

**DOI:** 10.1186/s13102-023-00728-x

**Published:** 2023-09-21

**Authors:** Sonakshi Sehgal, Aksh Chahal, Vandana Esht, Mohammed M. Alshehri, Rashid Ali Beg, Mohammad Abu Shaphe, Ramzi Abdu Alajam, Faizan Z. Kashoo, Ahmad H. Alghadir, Masood Khan

**Affiliations:** 1 Department of Physiotherapy, Career Point University, Kota, India; 2 Department of Physiotherapy, School of Medical and Allied Health Science, Galgotias University, Greater Noida, Uttar Pradesh India; 3 Physical Therapy Department, College of Applied Medical Sciences, Jazan University, Jazan, Saudi Arabia; 4 Department of Physical Therapy and Health Rehabilitation, College of Applied Medical Sciences, Majmaah University, Al Majmaah, Saudi Arabia; 5 Department of Rehabilitation Sciences, College of Applied Medical Sciences, King Saud University, P.O. Box. 10219, Riyadh-11433, Saudi Arabia

**Keywords:** Linear speed test, Agility t-test, Star Excursion balance test, Arrowhead change of direction test, Physical education

## Abstract

**Background:**

The intricate nature of an athlete's abilities evolves dynamically with the enhancement of motor skills. Hence the study sought to investigate the impact of a tailored four-week exercise program`encompassing exercises focused on balance, agility, and speed. The primary objective was to determine how this exercise program influences both the roller skating talent and overall physical fitness proficiency in young male roller skaters.

**Methods:**

Thirty male participants (age 11–14 years) enrolled in the school skating team were recruited. The participants were randomized into either an experimental group [*n* = 15], performing a short-term exercise program, or a control group [*n* = 15], involved in the physical education classes for eight sessions over four weeks. The primary outcome measure, the skating performance, was measured by the linear speed test (LST). The secondary outcomes, i.e., balance, agility, and speed, were evaluated using the star excursion balance test (SEBT), agility t-test (ATT), and arrowhead change of direction speed test (ACDT). SEBT was assessed in 8 directions. The study was registered with the Clinical Trials Registry India (TRN: CTRI/2018/09/015713) before the recruitment of the participants on 14/09/2018.

**Results:**

The results showed that LST, ATT, and ACDT improved significantly (*p* < 0.05) in both groups, however, greater (*p* < 0.05) improvement was observed in the experimental group (Cohen’s d 0.8 to 1.3). Regarding SEBT, improvement was observed in a few directions only in both groups. However, no significant difference was observed between both groups in SEBT measurements.

**Conclusions:**

A short-term structured exercise program consisting of balance, agility, and speed exercises significantly improved the talent of skating, agility, and speed compared to physical education classes activities in young male roller skaters. The study highlights the potential of targeted training interventions to enhance athletic performance in this population.

## Introduction

Talent identification and development programs have flourished across the globe. Athlete development has described talent as a result of inborn abilities or extensive experience at a young age. The factors such as relative age, birthplace, and socioeconomic status play an important role in the athletic development of high-performance sports and can affect skill development [1, 2]. The protocols for talent identification programs include the subjective, physiological, morphological, sociological, psychological, technical, and functional domains for evaluating athletes [3–5]. In the criteria of talent identification programs, the eligible athletes are chosen from the crowd. The criteria designed are such that the skills of the young persons are projected in their performance during adulthood [3]. In sports, talent is defined as the athletic abilities that consider the training experience and capabilities greater than the peer group of similar biological development, habits, and status [3, 6, 7]. The course of development of the athletes is hardly linear because cognitive and motor skills are intertwined and develop through dynamic interactions with the individual athlete’s performance environment [8–11].

Roller skating is a lifetime fitness sport for children and adults [12]. According to the available literature, balance, speed, and agility are efficiently required to perform well in every sport [13–16]. Improving skating performance requires understanding the contributing biomechanical variables [17]. Balance is the capacity to maintain the center of pressure on a support base with maximum stability and minor body sway [14]. Encouraging children to engage in physical activity can improve neuromuscular capacities and overall health [18]. Younger people with higher stability may benefit from the particular skating skills because skating is done on a relatively limited surface area, i.e., the blades that are in touch with a surface [19]. The sensory systems, such as visual, somatosensory, and vestibular systems that help with postural control and the motor systems that regulate muscular output, all undergo adaptations as a result of balance training [20]. Complex skating skills require a more refined balance, possibly getting better with training and maturity. The ability to quickly change direction and speed is known as agility [12, 21, 22]. As a result, it consists of two elements: direction modifications and stimulus-related decision-making [23]. In the scientific literature, it is recommended that agility be well-developed throughout childhood and adolescence as a crucial physical quality [24]. Visual processing, spatial awareness, dynamic balance, and rhythm are thought to be present in athletes with good agility [25]. The process of a child becoming good agile continues over a long period [12]. In many competitive sports, speed, especially forward linear speed, is one of the most crucial performance factors [26]. Speed is also considered an important element for maximizing performance on skates. The goal of the athlete during sprinting is to move from an initial point to the endpoint in the shortest time. Thus, it seems logical that training for maximum skating speed would correspond to training for sprinting [19].

To the best of our knowledge, there is a scarcity of research among pre-adolescent boys to determine roller skating talent after administering a structured exercise program. There are few pieces of research among similar age groups; however, these are associated with physical fitness components and talent development in soccer players, figure skaters, hockey ice skaters, and taekwondo practitioners [4, 15, 24, 27, 28]. Moreover, Enhancing a young skater's capacity to manage dynamic stance, agility and speed could be a crucial factor, especially considering that junior skaters undergo a phase of development marked by improvements in various physical capabilities. Hence, it is essential to analyze and train the parameters related to staking, aiming to enhance the competitive abilities of young skaters in terms of their technical skills. Therefore, following the above consideration, this randomized controlled trial aimed to determine the development or improvement of skating talent among young male roller skaters through an exercise program encompassing balance, agility, and speed exercises. The study also aimed at evaluating the improvement in balance, agility, and speed through this exercise program. It was hypothesized that the four-week-long exercise program consisting of balance, agility, and speed exercises would improve the talent of skating in young males.

## Materials and methods

### Study design

Two arms pretest–posttest experimental design was used to determine the effect of a structured exercise program, primarily on skating performance and secondarily on the balance, agility, and speed of young male skaters.

### Inclusion and exclusion criteria

Male participants of age 11–14 years in the skating team who were willing to participate were included in the study. Factors that could potentially impact skating or physical fitness skills, such as balance, musculoskeletal, or systemic disorders, were used as criteria for exclusion. Additionally, participants who had sustained recent injuries in the lower limb that made them incapable of participating in sports for more than seven consecutive days were also excluded from the study.

### Participants

**Fig. 1:**
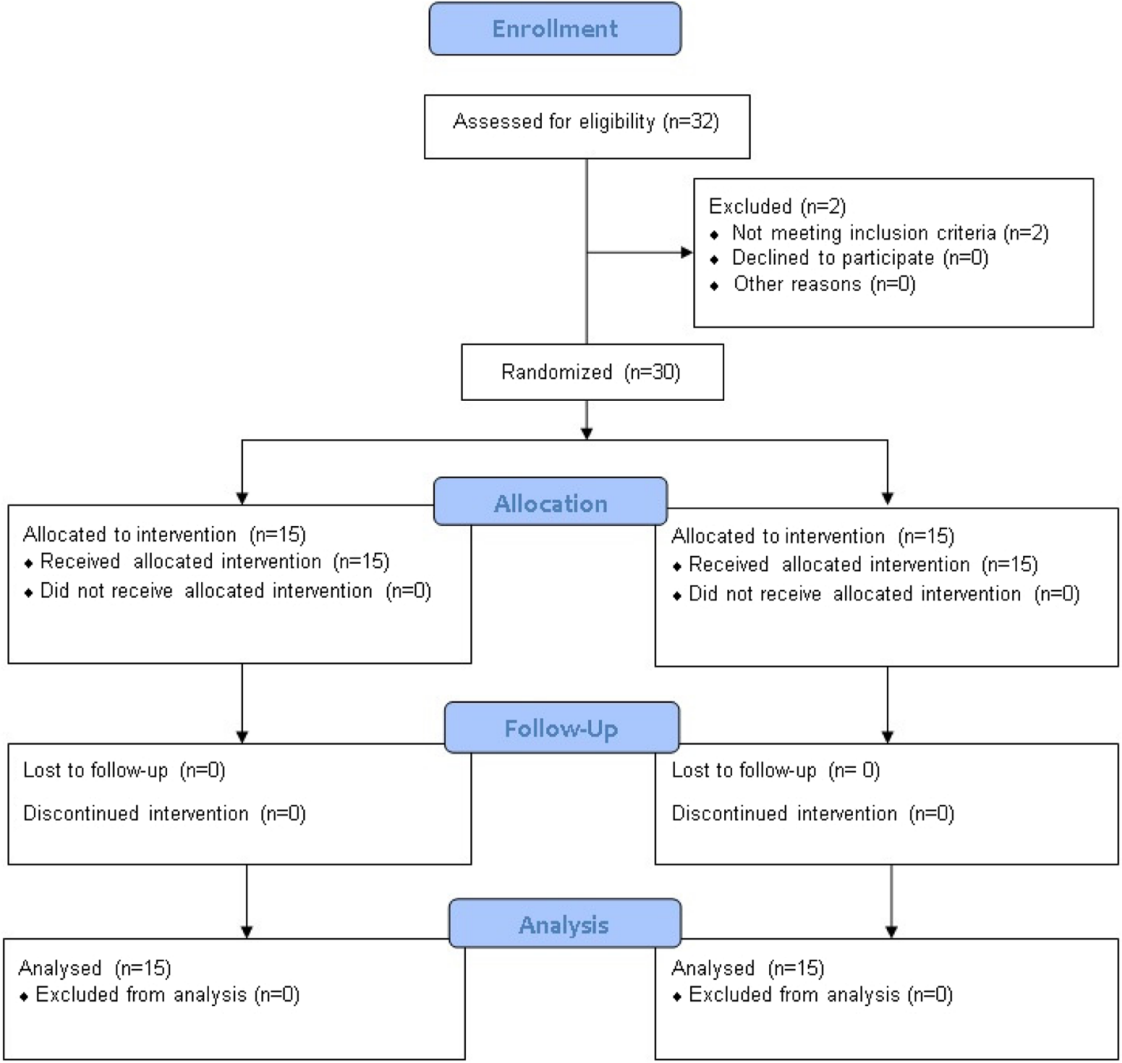
Consolidated Standards of Reporting Trials (CONSORT) showing the recruitment, inclusion, exclusion, randomization, and analysis of participants

Thirty-two male participants (aged 11–14 years) from a skating team at M.M. International School, Haryana, were assessed for eligibility. The study was performed in the Sports room of M.M. International School, India, from 1st October 2018 to 2nd November 2019. Among them, 30 participants were found to be eligible for enrolment in the study; two were excluded due to not meeting the inclusion criteria. Therefore, 30 participants were equally and randomly allocated to the Experimental group (EG) (*n* = 15) and Control group (CG) (*n* = 15) (Fig. 1).

The EG was assigned a structured exercise program of balance, agility, and speed exercises, and the CG followed their regular physical education classes under the guidance of their sports coach. The evaluation for skating performance, balance, agility, and speed was obtained before and at the end of the 4-week intervention. Demographic data, medical history, and anthropometric measurements were obtained. The study was registered with the Clinical Trials Registry India CTRI/2018/09/015713 before the recruitment of the participants on 14/09/2018. All methods were performed in accordance with the relevant guidelines and regulations. This study adhered to CONSORT guidelines.

All eligible and consenting participants underwent a baseline assessment of skating performance and physical fitness measures (balance, agility, and speed) before being randomized to the EG or CG by a physical educator who was not involved in the conduct or analysis of the trial. An independent researcher with the IBM SPSS Statistical software Version 20 generated a randomized sequence. The block randomization method was used to randomly allocate a total sample size of 30 participants into two groups. Randomization was done by 5 × 6 matrix design. There were random blocks of 5, with each block randomizing 6 participants. The participants were allotted to a group based on the generated randomized sequence. Once the block was allocated, the next row block sequence was generated. Thus, an equal number of participants were assigned to both groups over time. The participants were blinded to their group allocation using numbered, opaque sealed envelopes by an administrator who distributed them to participants sequentially as they completed their baseline assessment. The envelope was opened once the baseline assessment was completed. The outcome assessor was also kept blind to the allocation.

### Procedures

These exercises were incorporated into the protocol as in Table 1, titled, “Short term Exercise Program adapted from Exercise Protocol for Young Skaters” © Sonakshi Sehgal and copyrighted under the Copyright Office, Government of India with unique registration no: L-86552/2019 dated 25th October 2019. The exercise protocol is also given in our published protocol study [29].

**Table 1:** Short-term Exercise Program

Exercises	SETS (Reps/time)	Assisted Equipment
**Balance Exercises**		
Tandem walking (eyes open) Walking on toes	2 min	
Heel raise	2 sets (20 reps) with 30-s hold	
Toe raise	2 sets (20 reps) with 30-s hold	
Front slide, backslide, side slide on wobble board	20(each)	Wobble Board
Single limb squat	2 sets (20 reps)	
Single leg hop	2 sets (10 reps)	Marker (to mark the distance)
**Agility Exercises**		
Z -Drill	2 sets (3 reps)	Cones
T drill	2 sets (3 reps)	Cones
4 corner drill	2 sets (3 reps)	Cones
Figure of 8 drill	2 sets (3 reps)	Cones
**Speed Exercises**		
Running (30 m)	2 sets (3 reps)	
Speed ladder (8 m) Forward and side-to-side hop	2 sets (5 reps)	Speed ladder
10-yard sprint	2 sets	

### Intervention

All testing and training sessions were conducted at approximately the same time (9–10:30 a.m.) to avoid circadian variation. The participants were instructed to take a light meal an hour before the testing and maintain their normal activities, refrain from strenuous activity, or play an hour before the experimental sessions. Before the testing and exercise session, each test and exercise was explained and demonstrated to the participants to avoid unusual adaptations to the test or exercises. The movement quality was emphasized by providing verbal and visual feedback to the participants on exercise techniques. The exercise sessions were conducted on Wednesday and Friday of each week due to the availability of the participants on these days of the week. Before the exercise session, 10 min warm-up exercises were done, including jogging, vertical jumps, and a few stretches of the upper and lower limb general muscle groups. The participants were provided with cones, a speed ladder, and an area in a sports ground to perform all the exercises.

### The experimental group (EG)

Each 40 min exercise session consisted of balance, agility, and speed exercises [29], and a 2-min rest between each exercise session segment was performed. The training took place on Wednesday and Friday, with approximately a session lasting for 40–45 min. The exercises were performed in an orderly sequence of balance exercises, agility exercises, and speed exercises [29]. When introducing the exercise program to the participants, they focused on improving their balance skills, changing direction, and increasing their maximum speed during running. The details of the exercise program included in the study are given in Table 1. Intervention in this group lasted for four weeks.

### The control group (CG)

The participants in this group attended their regular physical education curriculum (also two times a week) for four weeks. They were primarily involved in skating and other low-organized games. No specific balance, agility, or speed exercises were taught to them during their physical education classes [29]. Participants in both groups were blinded to the study's aim to avoid any biased results between the two groups. The participants' adherence to the study was confirmed using the manual attendance maintained with the primary investigator.

#### Outcome measures:

1. Skating performance: The skating performance of the participants was evaluated using a linear speed test. A 30-m-long track was marked between a start line and a cone on a skating rink, and the participant stood at a start point of a 30 m long track. On a ‘Go’ command by the examiner, the participant skated linearly on the track to the finishing point with the maximum skating effort. Using a stopwatch, the examiner recorded the sprint time for covering a distance of 30 m [30, 31]. The test was performed for three trials, and the best trial was included. The trial was discarded if the participant fell during the test or did not complete the test with accuracy.
2. Balance performance: The balance was evaluated using the star excursion balance test (SEBT). The participants stood at the center of the grid of the lines joining at 45° from the center of the grid. The participants reach as far as possible with the unsupported leg in all eight directions. The reach distance was measured, and participants returned to bilateral stance. The examiner measured the linear distance manually from the center of the grid to the distance reached by the participant with a measuring tape in cms. When the right foot was used as a reaching foot, the balance of the left leg was evaluated. A clockwise fashion was followed when balancing on the left leg; similarly, an anticlockwise fashion was followed when balancing on the right leg. The test was repeated three times on each foot with 15-s rest in between each trial [32]. The best of the three trials in each direction was calculated. The trial was discarded if the participant lifted the stance foot from the center grid, lost balance at any point in the trial, or did not return to the starting position under balance [33]. The leg length was normalized with the excursion data by dividing each excursion distance by the participant’s leg length and then multiplying it by 100. Test results were recorded after each reach by the examiner.
3. Agility performance: The participants' agility was evaluated by an agility t-test. Three cones were placed in a straight line at a distance of 4.57 m, and the 4th cone was placed at a distance of 9.14 m from the middle cone to represent a figure ‘T’. On the ‘Go’ command by the examiner, the participant ran from the base of ‘T’ and touched the middle cone and ran to touch the left cone, then ran to the 9.14 m right cone and touched it, then again ran 4.57 m back to the middle cone and touched it and then ran 9.14 m back to the base of the ‘T’ and touched that cone [34]. The starting and finishing cones were the same. The examiner gave a command to ‘STOP’, and the test completion time was recorded using a stopwatch. Three trials were recorded, and the best trial was considered for evaluation. The trial was not included when the participant fell during the test performance or could not perform the test in a sequence [34].
4. Speed performance: The speed was evaluated using an arrowhead change of direction speed test. Three cones were placed in the shape of an arrow in a line at a distance of 5 m from each other, and the middle cone was placed 10 m from starting cone. The fourth cone was placed left of the starting point. The participant ran with maximum effort from a starting point (50 cm) to the middle cone, then ran to the left corner cone, then turned around and reached the extreme cone, and finally ran back to the point of start [35]. The time for completion of the test was recorded using a stopwatch. The best of the three trials was recorded for evaluation. The trial was discarded if the participants fell or failed to perform the test in a sequence [35].

### Data analysis

The sample size was calculated using G power 3.1.9.4 software (Heinrich-Heine-Universität Düsseldorf, Düsseldorf, Germany; http://www.gpower.hhu.de/). We determined 30 samples after adopting an effect size of 1.0 for the primary outcome [29], with the significance level set as 0.05 and the power set to 80%. The samples were randomized equally into two groups, with 15 participants in each group.

Data were expressed as mean and standard deviation, with the significance level set as *p* < 0.05. The normality and homogeneity of the data were confirmed by the Shapiro–Wilk normality test (*n* < 50). For the within-group comparison of skating performance and physical fitness tests, paired t-test was used. The mean change score (EG_means change_—CG_mean change_) was used to verify the difference in training effect between the groups through an independent t-test. The effect size (ES) was calculated to assess the standardized change in the mean values for pre and post-intervention scores for each group, using the formula, Cohen’s *d,* ES = (M post – M pre)/SD pooled, where M post is the mean post-intervention measure, M pre is the mean pre-intervention, and SD pooled is the pooled standard deviation of the pre-and post-intervention. Values of effect sizes < 0.2, < 0.6, ≥ 0.8 were interpreted as small, moderate, or large, according to Cohen [36].

## Results

Data from 30 participants were analyzed. The detailed demographic characteristics, including age, weight, height, BMI, and leg length of both group participants, are displayed in Table 2.

**Table 2:** Demographic characteristics of the participants in both groups and p-values for the independent t-test

Variables	EG	CG	*p*-value
	**Mean (SD)**	**Mean (SD)**	
Sample	15	15	
Age (years)	12 (1.1)	12 (1)	1.0
Weight (kg.)	50 (14.1)	55.6 (16.6)	0.373
Height (cm.)	149.7 (10.6)	152 (10.4)	0.5
BMI (kg/m^2^)	22 (4.5)	23.6 (4.6)	0.3
Leg length Right (cm.)	82.6 (7)	88 (6.7)	0.04*
Leg Length left (cm.)	82.5 (6.7)	87.7 (6.8)	0.04^*^

*EG* Experimental Group, *CG* Control Group

^*^Significant

### Primary outcome

LST: The analysis of skating performance measured using a linear speed test is presented in Table 3. The mean between-group difference in the linear speed test was -1.3 s (95% CI -1.8 to -0.7), t (28) = -0.2, *p* < 0.001). Although the skating performance improved in both groups, the change score between the group favored the EG by indicating a reduction of 1.3 s than in the CG. The paired sample t-test indicated a mean within-group change score of -1.6 s ( 95% CI 1.1 to 2.1) and -0.3 s (95% CI 0.02–0.6) in the EG and CG, respectively, which indicated a worthwhile improvement in the EG. A large effect (0.8) was reported in the EG for a linear speed test, while a small effect (0.1) was found in the CG.

**Table 3:** Mean (SD), with-in and between-group differences in the LST, ATT, and ACDT

Outcomes	Groups				Difference within-group		Difference between- group	Between-group *p*-value
	**Pre**		**Post**		**Post–Pre**		**Post–Pre**	
	**EG**	**CG**	**EG**	**CG**	**EG**	**CG**	**EG-CG**^b^	
LST	7.20 (2)	7.4 (2.2)	5.5 (2)	7.1 (2.2)	-1.6^*^ (0.8)	-0.3 (0.5)	-1.3 (-1.8 to-0.7)	< 0.001^a^
ATT	13.7 (2)	14.6 (2.4)	10.9 (2.2)	14.1 (2.2)	-2.8^*^ (1.4)	-0.4 (0.9)	-2.4 (-3.2 to-1.4)	< 0.001^a^
ACDT	11.5 (2.2)	13 (3.2)	8.9 (2.3)	12.5 (3.7)	-2.6^*^ (1.4)	-0.5 (0.9)	-2.1 (-3.05 to-1.2)	< 0.001^a^

*EG* Experimental Group, *CG* Control Group, *LST* Linear Speed Test, *ATT* Agility t-test, *ACDT* Arrowhead Change of Direction Test

^*^p < 0.005(Paired t-test)

^a^*p* < 0.001(Independent t-test)

^b^Negative between-group differences favor the experimental group

### Secondary outcomes

ATT and ACDT: Pre-intervention and post-intervention results of the Agility t-test (ATT) and arrowhead change of direction test (ACDT) are displayed in Table 3. For the ATT and ACDT, mean between-group differences were -2.3 s (95% CI -3.2 to -1.4) and -2.1 s (95% CI -3 to -1.2), respectively. That is, the mean difference favored the EG for ATT as well as ACDT. The mean difference reported a statistically significant result between the group for ATT (t (28) = -0.3, *p* < 0.001) and ACDT (t (28) = -0.3, *p* < 0.005). The ATT and ACDT within-group change scores showed improvement in the EG for ATT, 2.8 (95% CI 2 to 3.6) sec vs. 0.4 s (95% CI -0.02 to 1) and ACDT, 2.6 (95% CI 1.8 to 3.4) vs. 0.5 (95% CI 0 to 1). A large effect (1.3 and 1.1) was reported in the EG for ATT and ACDT, while a small effect (0.2 and 0.1) was found in the CG.

SEBT: Performance on the pre-intervention and post-intervention SEBT measurements within the EG and CG revealed statistically significant differences in functional reach of the right limb for anterior, posteromedial, and medial directions (*p* < 0.05 for these three directions). Significant improvements (*p* < 0.05) were found during the functional reach of the left limb (right lower limb stance) in all seven directions, however, not in the medial direction (*p* > 0.05) (Table 4). The independent sample t-test results revealed no statistically significant difference in all eight directions of SEBT between the EG and CG’s mean change scores (*p* > 0.05).

**Table 4:** Mean (SD) within and between-group differences in SEBT for right and left limb (normalized to reach distance)

**Direction**		**Right Leg Reach**		**Left Leg Reach**	
		**EG**	**CG**	**EG**	**CG**
ANT	Pre	93.6(16.7)	85.1(14.7)	94.9(13.3)	86.4(11.9)
	Post	94.6(16.4) ^*^	86.1(14.5) *	95.8(13.4) *	87.4(11.6)*
ALAT	Pre	92.4(16.7)	83.9(14.7)	93.7(13.3)	85.3(11.8)
	Post	94.6(16.4)	86.1(14.5)	95.8(13.4) *	87.4(11.6)*
LAT	Pre	91.2(16.6)	82.8(14.6)	92.4(13.2)	84(11.8)
	Post	91.4(16.7)	83(14.6)	92.6(13.3)*	84.3(11.7)*
PLAT	Pre	85.5(16.9)	79.4(18.9)	81.9(18.6)	82.4(17)
	Post	85.8(16.6)	79.6(19)	82.2(18.6)*	82.6(16.9)*
POST	Pre	84.3(16.6)	78.2(18.9)	80.7(18.6)	81.3(16.9)
	Post	84.8(16.8)	79.5(18)	81.2(18.6) *	82.5(16.4)*
PMED	Pre	86.7(17.6)	82(16.9)	86.8(19)	80.6(17.4)
	Post	87.1(17.5)*	82.4(16.9)*	87.1(19)*	80.8(17.8)*
MED	Pre	84.3(17.6)	79.9(16.8)	84.4(17.6)	80.2(17)
	Post	84.5(17.6) *	80(16) *	84.5(19)	78.5(17.6)
AMED	Pre	85.5(17.6)	81.1(16.8)	85.6(19)	79(17.7)
	Post	85.8(17) *	81.3(16) *	85.9(19) *	79.7(17.6)*

*ANT* Anterior, *ALAT* Anterolateral, *LAT* Lateral, *PLAT* Posterolateral, *POST* Posterior, *PMED* Posteromedial, *MED* Medial, *AMED* Anteromedial, *EG* Experimental Group, *CG* Control Group

^*^Significant (*p* < 0.05)

### Adherence to the study protocol

All the participants in the EG completed the scheduled exercise sessions each week. Each participant attended the post-intervention assessments, so there was complete data available for the outcomes. The exercise program was feasible and enjoyable for the participants in the EG, and no adverse events were reported during the sessions.

## Discussion

To the authors’ knowledge, this is the first randomized controlled trial that investigated the development of roller skating talent in young participants, using a structured exercise program of 4 weeks consisting of balance, agility, and speed exercises, performed in a school setting. The within-group changes in skating performance, agility, and speed showed large positive effects in the EG (Cohen’s d 0.8 to 1.3). A similar improvement in balance (SEBT) performance was noted in the EG and CG. The study's main finding indicated that the exercises implemented for four weeks (8 sessions) based on balance, agility, and speed contributed moderately to a large improvement in the EG’s skating and physical fitness skills.

The mechanisms behind the promising improvement by adhering to the short-term exercise program in pre-pubertal males might be particularly associated with enhancements in physical competency, eventually leading to improved skating skills. The maturity status and level of expertise play a key role in identifying talent in young athletes. Including the school students as participants were the primary endowment of this study. The development of talent in roller skating was due to the pre-pubertal age group included in the study because the learning and execution of the complex skills of skating and adaptation to the imposed training are adaptive in this age group. Few studies have analyzed the talent in sports performance after the skill performance test in a particular sport [4, 27, 37]. Some studies have analyzed improvement in various physical fitness components after applying different interventions on skaters. Lida Zare Dizajdizi et al. [12] investigated the effects of 8-week core stability exercises on selected motor skills and self-confidence in teenage roller skaters aged 14 to 16 years. Tina Winter et al. [14] evaluated the effect of 12 weeks of proprioceptive training on functional ankle stability in young speed skaters (12 ± 2.3 years). Although the two studies mentioned above were done on skaters of age groups similar to our study, they had a more extended training period as compared to our study, and in addition to that, they did not analyse the improvement in the skating skill.

In the present study, the skating performance was significantly improved in the EG (1.3 s); on the contrary, the CG (0.3 s) skaters showed no remarkable change in the skating performance. This signifies that improved balance, agility, and speed will enhance skating skills in skaters. The largest improvement was observed in agility (2.8 s) compared to speed (2.6 s) and skating performance (1.6 s) (Table 3). Developing basic skills of agility, balance, and coordination through movement stimulus is necessary for skating and other sports, with the ultimate goal of developing skills in skating just before the growth spurt [38]. The improvement in the skills of agility and speed occurs as a result of a repetitive movement during a change of direction that improves the physiological and biomechanical characteristics. Motor programming and neuromuscular system adaptation to the Golgi tendon organ, muscle spindle, and joint proprioceptors improved due to agility training [39]. Agility training enhanced the basic skills like walking and running technique, change of direction, and jumps that improved the agility on skate and overall performance. Skating speed depends on two factors which are stride length and stride rate, which eventually need to be trained during running [19]. In the present study, the training program for speed has trained both aspects by including running, sprinting, speed ladder running, and hurdle jumps in the exercise intervention. William B. Haug et al. [40] concluded that 4-week dry land sprint training improves the skating speed on the ice. They concluded that the change was due to skill development and improved motor patterns with time. Speed training enhances skate sprint locomotion mechanics and body position time measures due to translating a learned skill into the sport's performance. This concept is similar to Farlinger and Fowles’s study [27], which used a plyometric training program to increase on-ice skating performance. The authors also agree that there is a thin line between training and skill; movement-specific training can remarkably achieve positive results in improving sports performance.

The result of the present study indicated a similar improvement in the balance in both EG and CG. The balance training program had a positive impact on the performance of the athlete in their respective sports. Children acquire the skills gradually, in phases, as the nervous and musculoskeletal systems develop to the point that enhances motor learning at a given level of movement intricacy [8]. It is believed that balance development in children of the pre-adolescent age group depends on multiple sensorimotor systems [9]. The pre-balance exercises like the toe stand, tandem stand, and heel stand are significant in developing balance in the pre-adolescent age group [10].

Skating has the potential to improve balance and strength performances because the ability to control posture during actual skate motion with changing center of gravity requires sensory-motor coordination that eventually develops better neural pathways and sensory-motor systems [11, 12]. It was not surprising that in the present study, balance (SEBT) did not show a significant difference between both groups; this could be because the participants of both groups were skaters, and additionally, there is evidence that the capability of balance training to transfer training-induced effects to sport-related activities is limited [13].

The trial has some limitations that need to be mentioned here. Firstly, the stopwatch was used to record the timing of the tests, which may have induced some human error. Secondly, the present study was single-centered and only lasted for four weeks with limited sessions in a week. Future studies can use randomized control trial designs to investigate the effects of a longer exercise program than those conducted in the current study (> four weeks) on several physical performance variables. The study can also be a multi-centered study enrolling skaters from different schools. The use of an advanced timing recording system could be encouraged to avoid human error and improve precision.

## Conclusion

A four-week structured exercise program consisting of balance, agility, and speed exercises improved skating performance, agility, and speed in young male roller skaters. Given the improvements in skating performance, balance, agility, and speed, the study would advocate introducing these essential movement competency skills in pre-pubertal children in the school curriculum. These fundamental skills should receive primary attention and be incorporated at an early stage within any comprehensive plan for cultivating long-term athletic potential. This approach aims to bolster the foundational movement proficiency among young athletes.

## Data Availability

The data associated with the paper are not publicly available but are available from the corresponding author on reasonable request.
